# Characterization of the first complete genome sequence of yellow fever virus (YFV) in Sierra Leone: Implications for public health

**DOI:** 10.1371/journal.pntd.0014354

**Published:** 2026-05-18

**Authors:** John Demby Sandi, Taylor M. Brock-Fisher, Tiangay Mariama Patience Sallay Kallon, Marietou F. Paye, Ibrahim Umaru Fofanah, Dolo Nosamiefan, Mohamed Saio Kamara, Aiah Joshua Teh, Alhaji Turay, Colby Wilkason, Ian Baudi, Christopher Tomkins-Tinch, Chelsea I’Anson, Elyse Stachler, Jonathan E. Pekar, Al Ozonoff, Daniel Park, Christian Happi, Pardis C. Sabeti, Donald S. Grant

**Affiliations:** 1 Kenema Government Hospital (KGH), Ministry of Health, Freetown, Sierra Leone; 2 School of Community Health Sciences, Njala University, Moyamba, Sierra Leone; 3 The Broad Institute of MIT and Harvard, Cambridge, Massachusetts, United States of America; 4 Department of Organismic and Evolutionary Biology, Harvard University, Cambridge, Massachusetts, United States of America; 5 Institute of Genomics and Global Health, Redeemer’s University, Ede, Osun State, Nigeria; 6 Institute of Ecology and Evolution, University of Edinburgh, Edinburgh, United Kingdom; 7 Department of Pediatrics, Harvard Medical School, Boston, Massachusetts, United States of America; 8 Department of Biological Sciences, Faculty of Natural Sciences, Redeemer’s University, Ede, Osun State, Nigeria; 9 Department of Immunology and Infectious Diseases, Harvard T.H. Chan School of Public Health, Harvard University, Boston, Massachusetts, United States of America; 10 Department of Clinical Sciences, Liverpool School of Tropical Medicine, Liverpool, United Kingdom; 11 College of Medicine and Allied Health Sciences, University of Sierra Leone, Freetown, Sierra Leone; Oregon Health and Science University, UNITED STATES OF AMERICA

## Abstract

Yellow fever virus (YFV), a mosquito-borne orthoflavivirus that causes severe hemorrhagic disease, is endemic in parts of South America and Africa, yet genomic data from Sierra Leone is lacking despite ongoing case-based surveillance. Using hybrid-capture metagenomic sequencing, we generated a complete 10,611 nt YFV genome (98% coverage) from an adult male patient who reported to the Kailahun Government Hospital with fever and muscle pain. Phylogenetic analysis assigned the genome to the West African II genotype via the YFV Nextstrain build. The Sierra Leone genome showed 57 substitutions, three of which were non-synonymous (NS2B: N79S, NS3: V515I, and NS5 (RdRp domain): A643V), relative to its most recent common ancestor with other genomes from Senegal and the Netherlands. Bayesian phylogenetics estimated the time to the most recent common ancestor with these genomes as January 14, 2001 (95% HPD: December 17, 1987 - April 28, 2009), potentially indicative of long-standing transmission within West Africa that has not been genomically characterized, rather than specific localization to Sierra Leone. Together, these findings underscore the need for expanded genomic surveillance to monitor YFV spread and evolution.

## Author summary

Yellow fever is a viral disease spread by mosquitos which commonly causes fever and muscle aches, and can cause severe disease. Despite previously reported cases of yellow fever in Sierra Leone, there has not been any genomic information available about the virus in this population. In this work, we describe the first genome sequence from a yellow fever virus infection detected in Sierra Leone, and using phylogenetic approaches, we show that it is most genetically related to yellow fever virus sampled from Senegal and a Dutch individual that had travelled to Senegal and The Gambia. Our work fills a critical gap in the understanding of yellow fever virus diversity in West Africa, and highlights the important role that genomic surveillance plays in understanding circulating viral diseases, especially in regions such as Sierra Leone.

## Introduction

Yellow fever virus (YFV) is the causative agent of yellow fever (YF), an acute mosquito-borne viral haemorrhagic fever disease endemic in several countries across South America, Central America, and Africa [1]. YFV is a member of the *Flaviviridae* family within the orthoflavivirus genus, and carries a single-stranded, positive-sense RNA genome approximately 10,862 nucleotides in length [1]. The virus is genetically diverse, with seven phylogenetically and geographically distinct YFV genotypes reported to date [2]. Within Africa, there are five circulating genotypes: two in West Africa, and three in East and Central Africa [3].

Despite the availability of a safe and effective YFV vaccine [4], YFV remains a re-emerging vector-borne viral pathogen of major global concern, with multiple outbreaks reported each year in Africa and South America [4,5]. A recent global estimate by Wang and colleagues suggests an annual incidence of more than 86,000 cases, most occurring in sub-Saharan Africa [1]. Most confirmed YFV cases in Africa occur among unvaccinated individuals, highlighting the continued impact of low routine vaccination coverage in high-risk regions [6]. In Sierra Leone, YFV is an epidemic-prone pathogen that is under case-based surveillance, and based on serological testing triggered by clinical presentation, five confirmed YF cases have been reported across the country over the past 20 years: one case most recently in 2023, two cases from an outbreak in 2011, and two cases from an outbreak in 2008 [7–9]. However, the absence of genomic data from Sierra Leone limits our understanding of relative transmission dynamics and how locally circulating viruses relate to the YFV clades circulating elsewhere in Africa.

Through the Sentinel program, a national pathogen-surveillance effort integrating clinical sample collection with standard and multiplex CRISPR-based detection and sequencing, we received a total of 2,057 clinical excess samples from individuals who presented to the Bo, Kailahun, and Kenema Government Hospitals, with fever from July 2024 to March 2025. We tested these samples for circulating epidemic-prone pathogens using the high-throughput Combinatorial Arrayed Reactions for Multiplexed Evaluation of Nucleic acids (CARMEN), a CRISPR/Cas13-based assay [10]. We then sequenced samples that tested positive by CARMEN for further molecular analysis. Here, we describe the molecular features of YFV detected in one sample among this testing, which produced the first complete YFV genome sequence obtained in Sierra Leone.

## Results

### Sierra Leone’s YFV genome is a member of the West Africa II genotype

We detected YFV in a single excess clinical sample of blood plasma from an adult male who presented to Kailahun Government Hospital in November 2024, with fever, joint pain, and muscle pain. The sample tested positive on the CARMEN CRISPR-based diagnostic platform and was selected for genomic analysis. Because this was a retrospective study, only limited clinical information was available, and follow-up was not possible once the patient left the collection area. Therefore, we were unable to determine the timing of the patient’s symptoms relative to sampling, their vaccination status, any recent travel history, or the outcome of their infection.

**Fig 1:**
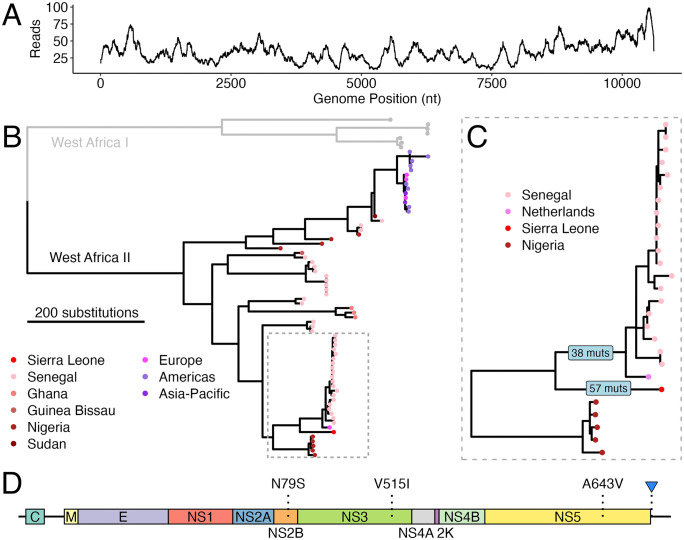
Genomic characterization of Sierra Leone's YFV genome. **(A)** Read depth profile across the 10,611 nt YFV genome generated from the Kailahun sample in Sierra Leone using hybrid-capture metagenomic sequencing. **(B)** Maximum likelihood tree showing the Sierra Leone YFV genome (red) in relation to a contextual dataset of 73 YFV genomes from the West Africa I genotype (grey) and West Africa II genotype (black). The Sierra Leone sequence clusters within the West Africa II genotype. **(C)** Expanded view on the clade containing the Sierra Leone sequence, highlighting its position as a sister lineage to genomes from Senegal and a travel-associated case detected in the Netherlands. Numbers on branches represent the number of mutations inferred along each branch. **(D)** Gene map highlighting the locations of the two non-synonymous polymorphisms consistent with reversion (NS2B: N79S and NS5 (RdRp domain): A643V) and one substitution (NS3: V515I) unique to the Sierra Leone YFV genome. The blue arrow in the panel also marks the 6-nucleotide region in 3’UTR that is deleted in the Dutch and Senegalese genomes but retained in the Sierra Leone and Nigerian genomes.

Based on a positive CARMEN result for YFV, we performed hybrid-capture metagenomic sequencing and generated a complete, 10,611 nt (98% coverage) YFV genome with an average read depth of 33.6 (range: 7–99; Fig 1A). Using the YFV Nextstrain build, the genome was assigned to the West Africa II genotype. We assembled a contextual dataset of 73 high-quality (>90% complete) YFV genomes from the West Africa I and II genotypes retrieved from NCBI and used these sequences to infer a maximum-likelihood phylogeny (S1 Table; Fig 1B).

Phylogenetic analysis revealed that the Sierra Leone genome formed a well supported (bootstrap support = 100) sister lineage to viruses recently detected in Senegal and a travel-associated case in the Netherlands who had reported travel in Senegal and The Gambia [11]. This combined cluster was most related to Nigerian samples collected between 2018 and 2020 (Fig 1C). Maximum-likelihood ancestral-state reconstruction in Nextstrain [12] indicated that these sequences likely descended from a Senegalese population (confidence = 86.7%), although the majority of genomes were collected in Senegal (S1 Fig; Fig 1B) which could potentially bias this estimation. The branch leading to the Sierra Leone YFV genome had 54 synonymous and 3 non-synonymous mutations, and the stem branch of its sister lineage had 33 synonymous and 5 non-synonymous mutations. Comparisons of the Nigerian, Dutch, and Sierra Leone YFV genomes revealed two non-synonymous polymorphisms consistent with reversion (NS2B: N79S and NS5 (RdRp domain): A643V) and one non-synonymous substitutions unique to the Sierra Leone genome (NS3: V515I). The Sierra Leone genome also retained the same nucleotide sequence at positions 10368–10373 of the 3’UTR as the Nigerian sample, rather than the 6-nucleotide deletion observed in the Dutch and Senegalese genomes (Fig 1D, blue arrow).

### Sierra Leone’s YFV genome started to diverge from the West African population around mid-January 2001

**Fig 2:**
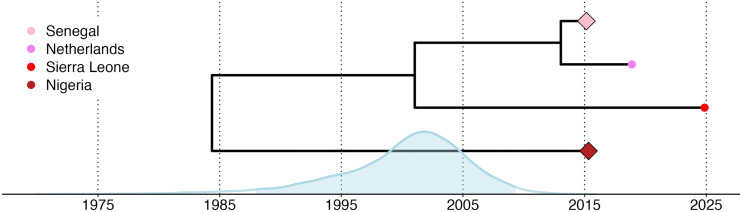
Time-scaled phylogeny inferred using BEAST X, showing the estimated divergence between the Sierra Leone genome and its closest related cluster of Senegal and Dutch genomes, as well as the Nigerian genomes. The time to the most recent common ancestor (tMRCA) is shown with the posterior height distribution, 95% HPD shaded in blue.

Bayesian phylogenetic analysis in BEAST X [13] estimated that the Sierra Leone genome diverged from its closest related Senegalese and Dutch sequences around mid-January 2001. The median inferred time to the most recent common ancestor (tMRCA) was January 14, 2001 with a 95 percent highest posterior density (HPD) interval from December 17, 1987 to April 28, 2009 (Fig 2). The estimated evolutionary rate across the full tree was 2.49e-4 substitutions per site per year (95% HPD:1.31e-4 - 3.63e-4), consistent with previous reports for sequences from the West Africa II genotype [14]. These results are consistent with an established lineage diverging from a larger West African population and persisting regionally, although they cannot rule out an introduction from another undersampled region. This uncertainty is further compounded by the lack of travel history for the patient and the overrepresentation of genomes from Senegal in the dataset.

## Discussion

Our genomic analysis supports sporadic serological data indicating YFV has been present in Sierra Leone for at least the past 20 years, although the limited availability of regional genomes constrains what can be concluded, and in particular we cannot rule out recent travel history of the patient described in this work. The placement of the Sierra Leone genome as a sister lineage to viruses from Senegal, together with a tMRCA in January 2001, is consistent with long-standing circulation within the wider West African region, although the specific geographic origin of this lineage cannot be determined from available data. Because YFV genomic data from Sierra Leone and neighboring countries are sparse, we cannot determine where this lineage circulated during the intervening decades or whether Sierra Leone was its primary reservoir. YFV ecology in West Africa also involves both sylvatic and zoonotic cycles, and cryptic circulation could reflect maintenance in wild non-human primates (NHP) or vectors rather than sustained human transmission. There have not been serological studies of YFV infection among NHP in the region, but modeling has suggested that conditions in Sierra Leone are favourable for serious transmission risk between NHP populations and human populations, and YFV antibodies have been detected elsewhere in Africa among NHP species that are endemic to Sierra Leone [15,16].

Our analysis identified two non-synonymous polymorphisms consistent with reversion to the genotype of the shared most recent common ancestor, as well as one substitution not observed in the next most closely related West African genomes, along with the retention of a 3′UTR region deleted in the Senegalese and Dutch sequences. At present, there is currently no evidence that these differences affect viral phenotype, and the functional significance, if any, of these differences remains unknown. Interpreting the impact of individual mutations is challenging without functional data, and future work will be needed to assess whether these differences influence viral fitness, vector interactions, or host range. Importantly, we did not detect mutations that have been experimentally linked to reduced vaccine neutralizing ability in mouse models [17], and there is no evidence from this genome to suggest altered vaccine sensitivity. This is consistent with epidemiological observations that most confirmed YFV cases in Africa occur among unvaccinated individuals and that current vaccines remain effective against circulating genotypes.

This study is subject to several limitations inherent to its retrospective design. We were unable to determine the patient’s vaccination status, clinical course, or specific risk factors for infection, as only limited metadata were available from this clinical excess sample. Although yellow fever vaccination has been routinely available in Sierra Leone since 2003, with reported national coverage exceeding 75% over the past two decades [7,18], we cannot assess whether vaccination status contributed to this individual case. Likewise, while the patient presented to a hospital setting, suggesting moderate to severe illness, we cannot characterize symptom progression or disease outcome. Although all available high-quality sequences were included in our contextual dataset, the dataset is unevenly sampled and enriched for sequences from Senegal, which may influence ancestral reconstruction. Finally, although we identified non-synonymous substitutions relative to closely related genomes, the functional impact of these mutations, if any, cannot be inferred without experimental data. Future work will be required to determine whether these substitutions influence viral fitness, vector competence, or host interactions.

Despite its limitations, the results of this study highlight the importance of strengthening surveillance for YFV in Sierra Leone and across West Africa, and in particular highlight the potential value of surveillance testing and sequencing after case detection. Routine testing for YFV in Sierra Leone is largely serological and based on those who present to clinics and hospitals. However, due to the similarity of symptoms between YF and other common infections, such as malaria or hepatitis B, testing may be under-utilized in clinical settings [7]. Further, while serological testing is useful for detecting exposure, it is insufficient for tracking viral diversity, origins, and movement. Integrating genomic sequencing into these clinical systems would provide critical complementary information to identify introductions, monitor lineage turnover, and evaluate potential evolutionary changes with relevance to diagnostics or vaccines. Additionally, testing among insect and NHP populations, along with increased surveillance testing of non-symptomatic individuals, would provide much better estimates on the broader prevalence of the YFV in the country and help inform public health measures to reduce spillover risks. Given the re-emergence of YFV across the region, expanding genomic surveillance, paired with ecological and epidemiological data, will be essential for enabling earlier detection, improving outbreak preparedness, and informing targeted public health responses.

## Methods

### Ethics statement

This work is part of a larger project, Surveillancing Circulating Pathogens in Sierra Leone, for which ethics approval was obtained from the Sierra Leone Ethics and Scientific Review Committee (SLESRC) (SLESRC No: 002/05/2024). No consent was obtained as the sample was clinical excess used only for research purposes and no identifying information about the participant was reported to the authors.

### Sampling technique/method

Clinical excess plasma samples collected from individuals who presented with fever at the Bo, Kailahun, and Kenema Government Hospitals were sent to the Kenema Government Hospital (KGH) viral hemorrhagic fever (VHF) research lab for a multi-viral pathogen screening as part of an ongoing diagnostic surveillance study. All samples were de-identified before transfer to the KGH VHF laboratory and were transported under cold chain conditions (2–8°C) using the standard triple packaging systems. Upon arrival at the KGH VHF laboratory, specimens were stored at −20 °C until analysis.

### Pathogen detection

Laboratory diagnosis of pathogens of public health concern was performed using a multiplexed polymerase chain reaction (PCR) approach combined with a Combinatorial Arrayed Reactions for Multiplexed Evaluation of Nucleic acids (CARMEN) CRISPR-based detection platform, as previously described [10]. Briefly, nucleic acids were extracted from 200 µL plasma samples using the Applied Biosystems MagMAX Pathogen RNA/DNA Kit (Cat #: 4462359) and subjected to a multiplex PCR amplification using in-house-designed pathogen-specific primer sets targeting the RNA-dependent RNA polymerase domain of the NS5 gene. Primer sequences and more details are available from [19]. Amplified amplicons were detected using CRISPR-Cas13, which allows for simultaneous detection of several targets in each sample on a Standard Biotools Biomark X instrument.

### Genome sequencing

Plasma samples in which at least one pathogen was detected by CARMEN were sequenced as previously described [20]. Briefly, we used the Illumina RNA Prep with Enrichment (L) Kit and the Viral Surveillance Panel (VSP) 2.0 to generate virus-enriched libraries following the manufacturer’s protocol. The libraries were then sequenced pair-end on the Illumina MiSeq platform.

### Sierra Leone genome assembly

We first demultiplexed the samples using the Broad Institute demux-deplete pipeline (github.com/broadinstitute/viral-pipelines/demux_deplete), followed by genome assembly using the Broad Institute assemble-denovo-metagenomic (github.com/broadinstitute/viral-pipelines/assemble_denovo_metagenomic) in Terra.bio using default settings.

### Dataset curation and maximum likelihood phylogenetics

We used Nextstrain [12] to search NCBI for YFV genomes that were at least 90% complete (length ≥ 9,775 nt). The resulting genomes were downloaded and Nextclade was then used to select only Clade IV and V sequences which correspond to West Africa I and II genotypes respectively, resulting in 73 contextual genomes (S1 Table). Complete genome sequences were then aligned to the vaccine strain reference (NC.002031) using augur align. A maximum likelihood phylogeny of these samples was generated with IQ-TREE v3.0.1 [21], using a GTR substitution model and 1000 bootstrap replicates. Ancestral state reconstruction and mutation annotation were completed with augur traits and augur ancestral respectively in Nextstrain. Trees were annotated and plotted using ggtree in R [22].

### Bayesian phylogenetics

From the complete genomic dataset described above, a subset of samples from the West Africa II genotype was selected to generate a time-scaled phylogeny using BEAST X [13]. We ran one chain of 100 million generations, subsampling every 10,000 steps to continuous parameter log and tree files, using a codon based substitution model (SRD06; HKY + gamma + 2) [23,24], an uncorrelated relaxed lognormal clock, and a flexible coalescent prior (a Bayesian skyride model, allowing for changes in population size through time [25]). Convergence was assessed using Tracer [26] and all ESS values were confirmed to be over 200. 10% burnin was discarded and trees were combined to an MCC tree using TreeAnnotater (https://beast.community/treeannotator) and visualized using ggtree in R.

## Supporting information

S1 Table Table of included contextual sequences including accession number, collection date, collection location, host name, genotype, and whether or not the sample is included in Fig 1C expanded view tree. (CSV)

S1 Fig Barplot showing the number of included genomes, per genotype, from each country. (TIF)
